# An assessment of American Indian women's mammography experiences

**DOI:** 10.1186/1472-6874-10-34

**Published:** 2010-12-15

**Authors:** Kimberly K Engelman, Christine M Daley, Byron J Gajewski, Florence Ndikum-Moffor, Babalola Faseru, Stacy Braiuca, Stephanie Joseph, Edward F Ellerbeck, K Allen Greiner

**Affiliations:** 1 Department of Preventive Medicine and Public Health, University of Kansas Medical Center, Kansas City, KS, USA; 2 University of Kansas Cancer Center, Kansas City, KS, USA; 3 Center for American Indian Community Health, University of Kansas Medical Center, Kansas City, KS, USA; 4 Department of Biostatistics & Informatics, University of Kansas Medical Center Kansas City, KS, USA; 5 Department of Internal Medicine, University of Kansas Medical Center, Kansas City, KS, USA; 6 Department of Family Medicine, University of Kansas Medical Center, Kansas City, KS, USA

## Abstract

**Background:**

Mortality from breast cancer has increased among American Indian/Alaskan Native (AI/AN) women. Despite this alarming reality, AI/AN women have some of the lowest breast cancer screening rates. Only 37% of eligible AI/AN women report a mammogram within the last year and 52% report a mammogram within the last two years compared to 57% and 72% for White women. The experiences and satisfaction surrounding mammography for AI/AN women likely are different from that of women of other racial/ethnic groups, due to cultural differences and limited access to Indian Health Service sponsored mammography units. The overall goals of this study are to identify and understand the mammography experiences and experiential elements that relate to satisfaction or dissatisfaction with mammography services in an AI/AN population and to develop a culturally-tailored AI/AN mammography satisfaction survey.

**Methods and Design:**

The three project aims that will be used to guide this work are: 1) To compare the mammography experiences and satisfaction with mammography services of Native American/Alaska Native women with that of Non-Hispanic White, Hispanic, and Black women, 2) To develop and validate the psychometric properties of an American Indian Mammography Survey, and 3) To assess variation among AI/AN women's assessments of their mammography experiences and mammography service satisfaction. Evaluations of racial/ethnic differences in mammography patient satisfaction have received little study, particularly among AI/AN women. As such, qualitative study is uniquely suited for an initial examination of their experiences because it will allow for a rich and in-depth identification and exploration of satisfaction elements.

**Discussion:**

This formative research is an essential step in the development of a validated and culturally tailored AI/AN mammography satisfaction assessment. Results from this project will provide a springboard from which a maximally effective breast cancer screening program to benefit AI/AN population will be developed and tested in an effort to alter the current breast cancer-related morbidity and mortality trajectory among AI/AN women.

## Background

Breast cancer is the most common malignancy among women in the United States and ranks second among cancer deaths in women [1]. As a result, early detection is key to survival. Results of United States (U.S.) and European randomized controlled trials demonstrate convincingly that regular screening mammography can reduce breast cancer mortality by up to 40% for women aged 50 and over. Mortality is strongly associated with staging of the cancer; women whose cancer is detected at earlier stages have better prognoses.

Breast cancer is more frequently diagnosed at a later stage among Hispanic and African American women compared to white women, which is likely due to their lower utilization of routine mammography [2,3]. Repeat use of mammography is beneficial in that several indicators of cancer prognosis (tumor size, axillary lymph node status, and stage) are favorably associated with cancers identified at subsequent screenings [4]. Smaller, noninvasive cancers are generally more amenable to treatment with breast-conserving surgery and are less likely to require systemic chemotherapy.

American Indians and Alaska Natives (AI/AN) have the poorest recorded 5-year cancer survival rates of any ethnic group and the lowest (or near-lowest) screening rates for major cancers [5]. Incidence rates have risen steadily over the last 50 years [6]. Breast cancer is the second leading cause of cancer death for AI/AN females [5]; and mortality rates among AI/AN women have increased, while decreased in other US ethnic groups [7]. Although breast cancer incidence is lower for AI/AN than other ethnic groups (58.0/100,000 women versus 89.8 to 140.8/100,000 women), 26% of AI/AN women with breast cancer will die from it. African Americans are the only ethnic group with higher breast cancer mortality [5].

Mammography is recommended annually for average risk women starting at age 40 [5]. AI/AN women have some of the lowest screening rates. Only 37% of eligible women report a mammogram within the last year and 52% report a mammogram within the last two years compared to 57% and 72% for eligible White women [5]. Reasons for this lag are unknown.

### Clinical Guidelines and Mammography Utilization

The American Cancer Society (ACS) recommends that all women 40 years of age and older obtain annual mammograms. Despite widely published clinical recommendations for screening by the ACS and other organizations, mammography rates remain suboptimal. Although mammography use has gradually increased within the past decade, data from the 2000 Behavioral Risk Factor Surveillance System survey indicate that only 22% of U.S. women aged 65 and older have had a mammogram within the past two years [8] and screening rates decrease as one ages [9]. In addition, although one-time and recent mammography use is high, adherence to guidelines (i.e., repeat mammography) is low [10,11]. Ethnic disparities in screening mammography also exist. Many studies indicate that African American and Hispanic women consistently use mammography services at rates below that of Caucasian women [12-15].

### Barriers to Mammogram Utilization

As a result of suboptimal breast cancer screening rates, identifying factors that affect routine mammogram utilization remains a national health priority. There has been no systematic ethnographic assessment of needs, barriers, knowledge, or attitudes to mammography in a heterogeneous AI/AN group, though the health disparities are well documented. Paskett and colleagues [16] surveyed Lumbee and Cherokee women in North Carolina and found higher barriers to mammography than White women from the same area, including "too hard to find time" and "no insurance". The survey did not use barriers named by community members. The authors called for research to identify ways to overcome barriers. Risendal and colleagues [17] surveyed knowledge, attitudes, beliefs, and behaviors in the Salt River and Guadalupe Indian communities and census tracts in the Phoenix area that contained high proportions of AI women. Barriers were not systematically examined, but factors predictive of having a mammogram in the past two years, included doctor recommendation, a Pap test in the last year, private insurance, availability of free or low-cost mammograms, knowledge of examination procedure, knowledge of recommendations, and worry about the results of the test.

Barriers to mammography have been studied in other groups, including African American [18-23], Latina [21,22,24], Asian [22,25], rural [18,26], and urban [18,21,24], with qualitative [19,21,24] and quantitative methods [18-20,22-26]. The barriers to breast cancer screening that have been discovered may be grouped into three categories: 1) physician-specific, 2) patient-specific, and 3) barriers encountered in the mammography process itself.

#### Physician-specific Barriers

Perhaps the most commonly cited barrier to mammogram utilization is a lack of physician recommendation for cancer screening [27-31]. Physician practices are extremely busy and patient acute care issues may take priority over preventive care. Studies indicate that organizational barriers, such as a lack of office reminder systems, plague the majority of physician practices [32,33]. Certain physician characteristics can also predispose physicians to recommend cancer screening to their patients. In a study of provider factors associated with higher repeat mammography rates, Burns et al. discovered that provider gender, type of practice (women's health group vs. internal medicine), and level of provider training of influenced repeat mammography in an urban medical center [34].

#### Patient-Specific Barriers

Many studies exist that have examined patient-specific barriers to mammography. Such barriers include issues related to age, race/ethnicity, socioeconomic status, education level, insurance coverage, and access issues. Despite the fact that a woman's risk for breast cancer increases with age, older women are less likely to obtain mammography services [35,36]. Highest utilization rates have been found among women in their 50 s [36-41]. In addition, minority women are less likely to utilize mammography services [36,42-47]. Findings from three studies indicate that low utilization among African American women may be attributed, in part, to fear of the mammogram procedure [15,46,48] and a reluctance to seek out or comply with medical advice [49]. Other studies report that Hispanic women have an even greater fear of cancer and its outcomes than do white and African American women [47,50].

Socioeconomic status also affects mammography utilization [51]. Poorer women are significantly less likely to get mammograms [52-54] as are women with fewer years of education [35,55]. Those who cannot afford health insurance coverage need to either pay out-of-pocket for mammography or qualify for programs that provide free access to mammography services. Even among the insured, co-pays or co-insurance can deter women from being screened routinely. For example, it is estimated that over 96% of persons 65 years of age and over receive services through Medicare [56], the majority of which have Part B coverage; under which mammography is a covered benefit. However, these women still pay a 20% co-insurance for mammography services, which can be a substantial amount for an older person who is on a fixed income, without supplemental insurance, and who does not qualify for Medicaid coverage.

### Access to Mammography

Access issues that may be classified as barriers to mammography include limited hours of operation, acceptance of self-referrals, ease of making an appointment, waiting room time, the use of reminder postcards or telephone calls, and location/type of facility. For the AI/AN population, this may also entail the pervasive unavailability of Indian Health Service practitioners and facilities. For example, all enrolled members of federally-recognized tribes are eligible for free health services at Indian Health Service (IHS) facilities and most AI/AN receive at least some of their care at IHS facilities. The IHS has 12 area offices overseeing 550 healthcare facilities across the US. The IHS does not have funding to place mammography machines in all clinics or to transport women to clinics that have them [57]. As such, many women go outside the IHS for screening.

A local IHS facility, Haskell Health Center (HHC) in Lawrence, KS (approximately 40 miles from the Kansas City Metropolitan area), serves approximately 8500 urban and rural (on- and off-reservation) AI/AN, and is administered by the Oklahoma City area office and staffed by a variety of medical professionals including physicians, nurses, pharmacists, and health education counselors. Their patient population represents over 200 AI tribes and over 30 AN villages. Although most patients live within 20 miles of HHC, some patients drive up to 70 miles for their medical services. HHC does not have mammography equipment. A third of their female patients are over age 40 and should, therefore, be having mammograms. A mobile unit from the Oklahoma office comes to HHC three days a year to serve these women.

### Satisfaction with Mammography

Patient satisfaction with mammography services is an important facet of mammography utilization. There is evidence from studies on patient satisfaction with medical care that dissatisfaction can lead to delay in medical treatment and nonadherence to treatment instructions [58,59]. In a survey of 255 women, Fine et al. found that more than 30% reported that their experiences with their first mammogram affected their decision to have subsequent mammograms [60]. Satisfaction is especially important in screening mammography where participants have no symptoms and are not motivated by ill-health to comply.

Published mammography patient satisfaction studies include self reports on the discomfort experienced during mammography [61,62], the efforts of health care personnel to ensure privacy, encouragement to ask questions, and the provision of information [63]. A longitudinal study of 6,898 women, found that unpleasant mammography experiences, such as enduring more pain than anticipated or being dissatisfied with the technique of the screening staff, was negatively correlated with return for future mammography [64]. In addition, a body of information is beginning to form regarding patient preferences for learning about the results of their mammograms. These studies have found that women prefer to be told about abnormal results by their primary care physician [65], and that satisfaction increases if results are directly interpreted so that further diagnostic studies can be performed while they are still at the facility [66,67]. These studies were conducted using instruments whose psychometric properties were not tested.

Two validated mammography patient satisfaction surveys have been developed. The first measure, developed by Loeken et al, is a 27-item instrument that emphasizes patient discomfort during mammography. This instrument includes eight subscales: physical discomfort, psychological discomfort (awkwardness, embarrassment), staff interpersonal skills, information transfer (explanation of procedure, comfort with asking questions), physical surroundings (ambience of waiting and examination rooms), future general satisfaction (advising others to have a mammogram), present general satisfaction (ensuring comfort), and staff technical skills [68,69]. The reliability, validity, and discriminatory power of this instrument have been examined with satisfactory results. All studies were conducted in Norway. Loeken and her colleagues subsequently used their instrument to compare patient satisfaction levels among six mammography facilities in Norway [70]. This study found considerable variation among facilities with respect to patient-reported pain, staff punctuality, information provided, and physical surroundings. To date this is one of the largest comparisons of patient satisfaction among mammography facilities.

The second measure, developed by Cockburn et al, is a 26-item instrument with six subscales: 1) convenience and accessibility, 2) staffs' interpersonal skills, 3) information transfer, 4) physical surroundings, 5) perceived technical competence of staff, and 6) general satisfaction. Cockburn's assessment of convenience and accessibility included waiting time, convenience of facility location, hours of operation. Questions on staff interpersonal skills included feeling hurried and politeness of staff. Information transfer questions covered explanation of the mammography procedure and an assessment of comfort with asking questions. Physical surroundings involved assessment of privacy and waiting room ambience. Questions on perceived technical competence were focused on mammography technologists. A general satisfaction subscale tapped into a woman's intent to return for screening. The reliability and concurrent and discriminant validity of this instrument have been examined. All studies on psychometric properties of this instrument were conducted in Australia.

Using their validated patient satisfaction questionnaire, Cockburn et al conducted a study to evaluate differences in satisfaction between persons attending an Australian screening facility for initial screening (N = 481) and recalls for additional views (N = 318) [71]. High satisfaction was found on most subscales among those attending the screening clinic while slightly lower satisfaction levels were reported in staff interpersonal skills, information giving, physical surroundings, convenience and accessibility, and general satisfaction subscales by those attending the recall clinic. Delays in reporting of results, however, were the source of most dissatisfaction among the recall clinic attendees.

Decker et al. [72] used a questionnaire developed by Cockburn [73] to examine differences in patient satisfaction by screening location and screening result following mammography. The setting included two screening facilities in Canada. The results of the study indicate overall high levels of satisfaction. Significant differences were found between the sites with respect to convenience and accessibility of the facilities and information transfer. In addition, women with abnormal findings were more likely to be dissatisfied with their mammography experience. This is a particularly important finding given that women with an abnormal result may be at increased risk for breast cancer. One drawback of this study was that 16% of the study sample (N = 1,176) completed the satisfaction question more than 12 months after their last mammography appointment at the facility, thereby introducing a recall bias.

An adapted version of Cockburn's instrument was also used in a study of 109 women who obtained mammograms at one of six different facilities. Dullum et al. determined that factors significantly associated with discomfort during mammography include patient perceptions of a technologist's "roughness" and the facility that a woman attended [74]. This study is also one of the largest comparisons of patient satisfaction among mammography facilities. In this study, however, it is unclear to what the facility-based differences may be attributed. For example, the differences may be due to technologist characteristics, differences in facility ambience, or issues related to patient access to the facility.

The studies above indicate different aspects of the mammography experience that patients perceive to be problematic. The Loeken and Cockburn patient satisfaction survey instruments, however, have only been tested in countries outside of the U.S. To our knowledge, no comprehensive evaluation of patient satisfaction (using a validated instrument) has been conducted within the U.S. The research proposed in this application is important, in part, because health care systems vary greatly not only by country but by geographic region, and it is likely that patient satisfaction with mammography will vary.

In the U.S., an accreditation program was established in 1994 by the Mammography Quality Standards Act (MQSA) with accreditation responsibilities being overseen by the federal Food and Drug Administration (FDA). Each U.S. mammography facility must adhere to an annual inspection process for continued certification. The inspection process addresses areas such as equipment, radiation dose, record keeping, quality assurance, and quality-control tests for film processors and for mammography and ancillary equipment. Included are initial qualification, continuing experience, and continuing education requirements for the various health care practitioners involved in the imaging process, such as physicians, medical physicists, and radiologic technologists. While this accreditation process is necessary to ensure that only high-quality mammography services are provided to women, its scope is limited. Current inspections do not review the total mammography facility environment and do not mandate quality improvement initiatives that examine and respond to patient satisfaction issues.

We do know that a woman's prior experience affects the likelihood of repeat mammogram utilization. It is clear that mammogram utilization is influenced by a person's racial/ethnic background. Racial/ethnic differences in mammography patient satisfaction, however, have received little study. Furthermore, such studies have not been focused on AI/AN women, a population whose breast cancer screening rates remain in the lowest range of those measured. The following research proposal describes how we will assess such ethnic disparities. For effective interventions to be developed for this population, the AI/AN woman's mammography experiences must be understood. Qualitative study is uniquely suited for an initial examination of their experiences because it allows participants to express and explain their experiences rather than rate them from a pre-existing list that may or may not include all relevant topics. This formative research is an essential step in the development of a validated AI/AN mammography satisfaction assessment and will provide a springboard from which a large-scale breast cancer screening program in a heterogeneous AI/AN population may be created.

This study was approved by the University of Kansas Medical Center Human Subjects Review Committee.

## Study Aims and Hypotheses

### Aims and Hypotheses

**Aim #1: **To compare the mammography experiences and satisfaction with mammography services of Native American/Alaska Native women with that of Non-Hispanic White, Hispanic, and Black women.

**Hypothesis**: There will be significant and describable variation in women's experiences and satisfaction with mammography experiences between American/Alaska Native, Non-Hispanic White, Hispanic, and Black women.

**Aim #2: **To develop and validate the psychometric properties of an American Indian Mammography Satisfaction survey.

**Aim #3: **To assess variation among American Indian/Alaskan Native women's assessments of their mammography experiences and mammography service satisfaction.

**Hypothesis**: There will be significant variation in satisfaction with mammography experiences between women receiving mammography services within the Indian Health Service system vs. those receiving services outside of the Indian Health Service system.

## Methods/Design

### Study 1: Formative Research: Assessment of Racial/Ethnic Differences in Mammography Experiences and Satisfaction

The majority of mammography patient satisfaction studies have been conducted using assessment instruments whose psychometric properties were not tested, have been tested outside of the United States, or have not focused on the at-risk American Indian/Alaskan Native population. In this project, we will develop and validate a mammography patient satisfaction instrument that will be tailored for use by American Indian/Alaskan Native women (See Table 1: Implementation Plan). As this is an understudied area, we believe that a study design utilizing qualitative techniques can offer insight into the issues surrounding AI/AN patient satisfaction with mammography [75]. To develop a culturally appropriate patient satisfaction assessment we will identify the mammography experiences and issues that affect the satisfaction of American Indian women through the use of focus groups based on ethnographic research methods. Ethnographic research is the traditional methodology of cultural anthropology and is used to learn about the social and cultural life of a population. It is a scientific and investigative approach with several characteristics including using the researcher as the primary tool of data collection, using rigorous research methods and data collection techniques to avoid bias and ensure accuracy of data, emphasizing and building on the perspectives of the people in the research setting, and using inductive techniques to build local theories for testing and adapting them for use both locally and elsewhere [76]. Ethnography uses both qualitative and quantitative techniques to elicit information from participants [77]. For this project phase, our primary data collection and analysis will be qualitative (focus groups). We will augment our focus groups with systematic quantitative data collection and analysis.

**Table 1:** Implementation Plan

Study	Tasks	Year
**Study 1: **Assess racial/ethnic differences in mammography experiences and satisfaction	Hire staff, conduct focus groups and comprehensive analysis of qualitative data from current project's American Indian focus groups and qualitative data from prior research team study with Non-Hispanic White, Hispanic, and Black women.	Year 1

**Study 2: **Development and testing of American Indian Mammography Services (AIMS) Survey	Develop survey and Healthy Living American Indian Women website and pilot test survey.	Years 2 - 3

**Study 3: **Assess variation in American Indian mammography satisfaction	Recruit American Indian women for AIMS study and analyze AIMS data	Years 2 - 5

We propose an in-depth, ethnographic analysis of mammography experiences in general and those leading to satisfaction and dissatisfaction with mammography services in a heterogeneous AI/AN population.

#### Focus Groups

We will conduct 12 focus groups in study 1. Focus groups, a standard tool in market research, have been employed by health researchers to obtain qualitative information for developing and testing various interventions and messages [78]. Focus groups are used to obtain in-depth information regarding awareness, feelings, attitudes, beliefs, fears, experiences, values, needs and reactions regarding issues or products.

##### Participants

Twelve focus groups, each comprised of 8-12 unacquainted individuals, will be conducted. Focus groups will be conducted with American Indian/Alaskan Native women and will be stratified by residential location of participants (e.g., rural or reservation vs. urban). Participants will include women who are 40 years of age and older (in concordance with the American Cancer Society's recommended beginning age for annual screening of asymptomatic women) and will be further stratified by age (e.g., 40-64 year-olds and those aged 65 years and older). All participants will have had at least one mammogram performed within the past five years.

Focus group participants will be recruited at an Indian Health Service clinic through referral from clinic personnel and through posters in the waiting area. Participants who do not use the above-mentioned health service clinic may be more difficult to reach because there is no central location through which to reach them. We will post flyers and posters at various community locations in the four primary Kansas Indian Reservation sites located in Horton (Kickapoo), White Cloud (Iowa Tribe of Kansas and Nebraska), Mayetta (Prairie Band Potawatomi Nation), and Reserve (Sac and Fox Nation) Kansas. We will also post flyers at other local AI/AN venues including the Heart of America Indian Center and the offices of the American Indian Council. A brief questionnaire at the time of recruitment will allow us to assign groups based on the sampling frame illustrated in Table 2.

**Table 2:** Focus Group Sampling Frame

AI/AN Women			
**Age 40-64**		**Age 65+**	

**Rural/Reservation**	**Urban**	**Rural/Reservation**	**Urban**

3 groups	3 groups	3 groups	3 groups

36 participants	36 participants	36 participants	36 participants

Focus group moderator's guides will be developed with input from program advisory board members and will be based partially on information from our prior mammography focus groups with Non-Hispanic White, Hispanic, and Black women. Discussions will focus on barriers to obtaining mammograms, motivating factors to obtain mammograms, past mammography experiences, and areas of mammography satisfaction and dissatisfaction.

##### Conduct

Focus group meeting times will be two hours and all groups will be audio- and videotaped. We understand that effective use of the focus group requires development of insightful, appropriate questioning frameworks and outlines for the group moderator. As a result, an American Indian moderator, informed of the study goals and operating with a prepared outline, will facilitate and elicit responses from participants. The moderator will take steps to create a nonthreatening, nonjudgmental, supportive climate conducive to open exchange and expression, ensure that the session direction remains relevant to the study, ensure that all group members are allowed to contribute, and ensure that responses are not inhibited or shaped by one or two dominant members. Prior to the start of the focus group, all participants will provide both written and verbal informed consent and then complete a brief demographic survey. Any person who does not wish to consent will not be eligible for participation. Participants in the focus groups will be reimbursed $25.00 for their time and transportation. We will also provide a light meal for all focus group participants.

##### Mammography Satisfaction Focus Groups with Hispanic, White, and Black Women Conducted Previously

We will pair the AI/AN focus group information ascertained within this project with transcripts from Dr. Engelman's prior focus group study with Non-Hispanic White, Hispanic, and Black women [79]. In brief, however, Dr. Engelman conducted focus groups with over 100 women to identify issues related to mammography satisfaction. These groups were held in rural and urban communities and were conducted with African American, Hispanic, and Non-Hispanic white women. Focus group participants identified five major themes that contributed to satisfaction with their mammography experiences: 1) scheduling appointments, 2) mammography exam discomfort/pain, 3) mammography facility environment, 4) treatment by the mammography technologist, and 5) reporting of mammography results. Further analyses have also been conducted to assess differences between racial/ethnic focus group participants. These analyses found pain and waiting room time to be issues that primarily affected African American women. Hispanic women largely experienced embarrassment and fatalistic beliefs during mammograms. White women were most affected by instructions that either prepared them for the exam or were given during the exam. The transcripts from these focus groups conducted for another study will be re-examined according to the current study rubric described above. The availability of these transcripts and pairing of them with the current study focus group transcripts will allow for a rich assessment of the variations in mammography experiences and satisfaction between women belonging to four very distinct racial/ethnic groups and shed particular light onto the mammography service issues that are of particular importance to each population group. These data will provide necessary insight into areas of mammography service that may be tailored to women from differing racial/ethnic background to enhance their mammogram experience.

##### Qualitative Data Analysis

Transcribed audiotapes will be coded and analyzed using ATLAS ti, a qualitative data analysis program. The coding protocol will be developed using a combination of several qualitative analysis approaches. Initially transcriptions will be "open coded": identifying within the text key words, themes, and descriptions of behavior [80]. Subsequently, we will group these themes into coding categories and develop a code map. This will allow us to categorize and retrieve issues related to patient satisfaction, barriers to obtaining mammograms, mammogram preferences, and culturally-specific issues related to obtaining mammograms. Key ideas, words, phrases, and recommendations will be examined to formulate summary statements and conclusions. For core coding categories, two independent observers will code data. We plan separate analyses for each stratum to see if differences emerge. In addition to text analysis, we will develop ethnographic decision models (EDMs) for each stratum. EDMs are causal models that predict the types of choices people will make under certain circumstances [77]. They have been used in anthropology for many purposes, including the way people decide which treatment to use. In this case, EDMs will consist of decisions a woman must make to decide if she will have a mammogram. EDMs will be evaluated to develop a comprehensive EDM for the population which will help develop an effective intervention to increase mammography rates in AI/AN women.

### Study 2: Development and Validation of an American Indian Mammography Services (AIMS) Survey

The majority of mammography patient satisfaction studies have been conducted using assessment instruments whose psychometric properties were not tested, have been tested outside of the United States, or have not focused on the at-risk American Indian/Alaskan Native population. In this study, we will develop and validate a mammography patient satisfaction instrument that will be tailored for use by American Indian/Alaskan Native women. To develop a culturally appropriate patient satisfaction assessment we will identify the mammography experiences and issues that affect the satisfaction of American Indian women through the information gleaned from the focus groups performed with AI/AN women for this study as well as Dr. Engelman's prior focus groups with Non-Hispanic White, Hispanic, and Black women.

#### Development of an American Indian Mammography Satisfaction (AIMS) Survey

A mammography patient satisfaction survey was developed originally in 1991 by Cockburn and her colleagues [73]. This instrument includes 6 subscales: 1) convenience and accessibility, 2) staffs' interpersonal skills, 3) information transfer, 4) physical surroundings, 5) perceived technical competence of staff, and 6) general satisfaction. The reliability and validity of this instrument has been tested through research conducted in Australia. Dr. Engelman's mammography workgroup has also developed the Patient Assessment of Mammography Services (PAMS) mammography satisfaction for use with the general U.S. population. The PAMS survey assessed mammography satisfaction in the following areas: appointment scheduling, facility environment, exam, mammography technologist, mammography results reporting, and overall satisfaction. An additional open-ended question asked women to provide suggestions for improving the mammography experience. The Cockburn et al. and PAMS instruments will serve as the basis for our culturally tailored American Indian Mammography Satisfaction (AIMS) survey. We intend to modify and supplement these instruments by including questions that relate to the main themes and ideas generated through the focus groups that are not included in the Cockburn instrument. We will categorize questions into subscales and anticipate that additional subscales, beyond those included in the Cockburn instrument and our research team's PAMS survey, will include greater detail on facility access, hours of operation, acceptance of self-referrals, ease of making an appointment, location/type of facility, the use of reminder postcards or calls to patients, or the mode and timeliness of reporting exam results to patients. We will calculate patient satisfaction scores by numerically assigning each possible answer and summing answers within each scale. A global satisfaction score will be obtained by summing all subscale scores. We will also collect demographic data on participants' date of birth, education, marital status, income, employment status, race/ethnicity (e.g., American Indian, Alaskan Native, or mixed race), the number of mammograms obtained in the past, and history of breast malignancies. Our goal is to develop a brief instrument that can be administered easily.

##### Internet-Based Health Information

More health consumers are using the Internet for information than ever before, including lower-income, less educated, and minority Americans [81]. Studies show health-seeking Internet use among low-income AA [82], low-income AA parents [83], White parents [84], AI [85], and Latinos [86]. However, there is a persistent gap in computer and Internet use between the majority population and ethnic minorities, low-income, and rural populations, though there are large increases in computer ownership and Internet use over time among all demographic groups [87]. Overall, Whites are more likely to use the Internet than Latinos and Latinos are more likely to use the Internet than AA [88]. AI have not been compared to other ethnic groups [85]. Patients are likely to share the information they learn online with providers, find the information useful, and make changes based on it [82-84,89]. Therefore, information must be accurate and appropriate for the people using it, in terms of culture, literacy, and ease of use or navigation [81,87,90-93]. Trends in the digital divide may be changing, as the gap between the majority population and others is decreasing and there has been a significant increase in the public availability of computers and Internet access at schools, public libraries, and workplaces [94]. Our own studies among AI show a strong interest in using the Internet for health information, an acceptance of using computers in public places, and a preference for culturally-tailored applications and information. In her prior studies with the American Indian population, Dr. Daley has found success with the use of Internet-based surveys. As such, the AIMS will be designed as an instrument that is completed by participants via the Internet (a corresponding paper survey will also be available for those with a preference for paper-based surveys).

#### Internet-Based AIMS Survey (Programming and Development)

##### Comprehensive Research Information System (CRIS)

The Internet-based AIMS survey will be made available through a state-of-the-art Clinical Information Management Database System that supports a Comprehensive Research Information System (CRIS). The database system is a secure, 21 CFR Part 11-compliant, robust and scalable system such that data can be entered efficiently and in a standardized format. This web-based system allows for direct data entry from participants. Among other things, the comprehensive database management system supports participant recruitment, study monitoring, data safety monitoring, and query management.

Our Comprehensive Research Information System provides capabilities to: 1) create, maintain, and edit participant data; 2) track study protocol versions, amendments, and IRB approvals/renewals; 3) create participant screening and enrolling criteria; 4) create and disseminate case report forms for outcomes studies; 5) create participant schedules and record participant status; 6) create user and multi-organization research networks; 7) record, maintain, and report adverse events; 8) conduct study queries and generate standard and ad hoc reports; and 9) export clinical data to third party analytical tools, such as SAS and Microsoft Excel. In addition, the system will allow for real-time monitoring of participant survey completion and patient data with built-in data quality, auditing, and integrity checks. The system will provide our researchers with a central repository for all study related documents and also will allow for the automation of research administration activities.

##### Healthy Living American Indian Women Internet Portal

The Healthy Living American Indian Women Internet portal will be developed as a separate website from the actual CRIS database. The Healthy Living American Indian Women Internet portal website will be linked with the CRIS database so that any survey completed within the Healthy Living American Indian Women Internet portal is transmitted back to the CRIS database and resides there under that specific participant information. This is how we will protect the integrity and security of the data collected. The AIMS survey participants will not have access to the actual CRIS database and they cannot alter the AIMS survey housed on the Healthy Living American Indian Women Internet portal website, they can only fill it out and submit it.

##### Survey Form Development

Through previous collaborations with various clinical research groups, the Center for Biostatistics and Advanced Informatics (CBAI) staff has over 40 years collective experience in the design, development, validation and implementation of survey forms that are consistent with protocol, reporting and various sponsor requirements. The design of the AIMS survey will be collaboration between the Clinical Information Specialists of the CBAI, the project director (Dr. Ndikum-Moffor), the Principle Investigator (Dr. Engelman), and the study statistician (Dr. Gajewski). Once all variables of interest have been identified and verified with the study endpoints and proposed analysis, the initial drafts of the AIMS survey will be designed in the development environment of the comprehensive research information system. Once the AIMS survey is created, it will be reviewed for completeness, accuracy and utilization by study staff and expert reviewers. Mock AIMS surveys as well as AIMS pilot testing will be completed to ensure compatibility with the data being collected in the source documentation as well as used for validation of the range, logic and other edit checks in the web-based data entry system. Once the AIMS survey is validated and final, it will be locked to format and editing in the system and added to the production environment for remote data entry.

Women who express interest in participating in the study will be assigned a unique identifier upon their first visit to the site. This ID will allow participants to save their AIMS survey in progress and return later for completion. Each participant will be provided with directions for the location of the AIMS web-survey: once she is at the website, the participant will have to enter her unique ID and a password to access the AIMS survey.

##### Web-Survey Technical Difficulties

Using a web application will ensure that all interested women will be able to access the AIMS survey. While most women will likely participate using laptop computers made available by the research team at recruitment events, a number of others will be more comfortable with accessing and completing the AIMS survey in the privacy of their own home. The minimum requirements will be a computer running an Internet Explorer version 4.0+ or equivalent and access to the internet. This includes more than 99% of all computers (browser usage from http://www.thecounter.com/stats/). Women who forget or lose their unique ID and password will be addressed by having users identify and answer "secret" questions upon their first visit to the site (e.g., First pet's name?). This information will be stored separate from study data. A participant who forgets her ID will be able to retrieve it by answering the questions she identified and answered.

##### AIMS Content Validity

We will use American Educational Research Association, American Psychological Association, and National Council on Measurement in Education's *Standards for Educational and Psychological Testing *released in 1999 to guide our instrument validation process. Goodwin provides a summary of these validation standards [95]. During our AIMS pilot testing phase, we will first garner evidence for instrument content. A panel of nine content experts from the fields of breast screening, cancer control, and American Indian health will evaluate the items and instrument according to the procedure set forth by Grant & Davis [96]. They will receive a content review form divided into domains, each with an accompanying definition, and will be asked to rate the items' relevance to the domain on a 4-point scale, (i.e. from 'not relevant' to 'highly relevant'). We will drop, revised, or kept items based on responses across experts, using the item content validity index [97]. The instrument will undergo a final review for readability and design by the project investigators. Additionally, we will use content information to re-calculate sample sizes for participant portion of this study if necessary.

#### AIMS Pilot Test

We will conduct mock tests of the AIMS Internet instrument and analyze the questions for logistical problems such as skip patterns, range errors, and response issues. Once all logistical issues are resolved, we will pilot the AIMS Internet survey with a sample of 50 American Indian/Alaskan Native women. The pilot testing procedure described below will allow us to administer the AIMS to a sample of women to determine if the questions are presented in a clear and understandable manner, assess the face validity of the questions, and evaluate the survey administration procedure. The pilot test will help guide the final refinement of the AIMS.

After completing the AIMS web survey, additional questions will be tagged on the end of the assessment that inquire about: 1) whether participants felt the survey questions clearly assessed the important aspects of mammography services, mammography satisfaction and dissatisfaction, and barriers to mammography utilization, 2) whether participants felt comfortable responding to all of the questions, 3) perceptions regarding the clarity and completeness of the information included in the AIMS survey, 4) the personal relevancy of the survey topics, and 5) the readability/presentation of the AIMS survey. Survey items will be revised based on feedback and re-piloted with additional participants until an acceptable AIMS survey has been developed.

##### AIMS Pilot Testing Recruitment

AIMS pilot testing participants will be recruited at the Haskell Health Center through referral from clinic personnel and through posters in the waiting area. Participant eligibility criteria are outlined in Table 3.

**Table 3:** Participant Eligibility

Inclusion Criteria	Exclusion Criteria
• Aged ≥40 years of age • Mammogram within past 5 years • Last mammogram at IHS or non-IHS facility • Home address & access to a working telephone • American Indian/Alaskan Native • Female	• Cognitive impairment • Another household member enrolled in the study • High risk for breast cancer

##### Pilot Test Compensation

To compensate for their time, we will provide each participant with a $25 gift card for completing and commenting on the AIMS survey.

### Study 3: Assessment of Variation in American Indian Mammography Satisfaction

#### Full AIMS Implementation Procedure

##### AIMS Recruitment

As with the focus group and AIMS pilot testing portions of this study, AIMS survey participants will be recruited at the Haskell Health Center through referral from clinic personnel and through posters in the waiting area. We will also recruit at 4-5 AI/AN community events held each year (one event in each of the four Kansas-based reservation cities (e.g., Horton, White Cloud, Mayetta, and Reserve, KS). These community events will include health fairs and Pow Wows. This multi-method plan of recruitment will allow us better access to participants who do not use IHS facilities for mammograms. In advance of events, we will post flyers and posters at various community locations in the four primary Kansas Indian Reservation sites and at the Haskell Health Center. We will also post informational flyers at other local AI/AN venues including the Heart of America Indian Center and the offices of the American Indian Council. We will make two Internet-ready laptop computers available at each community event so that interested participants readily may complete the AIMS survey. Postcards with the Healthy Living American Indian Women website address will be provided to those women who express interest but who prefer to complete the assessment in the comforts of another environment (e.g., their home or local library). An additional paper and pencil version of the AIMS survey will be available on site at community recruitment events for those individuals preferring a non-electronic information submission approach.

The Principle Investigator and Project Manager will maintain close contact with key community member stakeholders involved in the proposed recruitment venues and will conduct ongoing assessments of the feasibility of the procedure used to identify women for the AIMS survey. Appropriate adjustments in the recruitment protocol will be made to meet survey accrual needs.

Eight to 10 subjects per AIMS survey item will be used during this study (for example, if 25 items are included in the instrument, 200-250 patients will be used). We will mail postcard reminders and telephone persons who were recruited and expressed an interest in participating in the AIMS on their own time but who fail to engage with the survey to maximize recruitment efficiency and survey completion.

#### Data Analysis

##### Statistical Analysis

We will use means and standard deviations to summarize quantitative demographic information and frequencies. Percentages will be used to summarize categorical demographic information of the participants surveyed.

##### Internal Consistency

Data will be analyzed for internal consistency within domains to demonstrate the appropriate clustering of items. Each domain will be individually assessed using Cronbach's alpha, the standard statistical technique for assessing the coherency of each item within each domain.

##### Concurrent Validity

Concurrent validity is defined as the degree to which the scores on an instrument are related to the scores on another instrument administered at the same time, or to some other criterion variable at the same time. We will assess concurrent validity by correlating the sum of the subscale scores for each respondent with their score on the general scale.

##### Construct/Discriminant Validity

Construct validity is the degree to which an instrument measures an intended construct. Multiple regression will be used to examine the discriminant validity of the proposed subscales and possible support for the multidimensional conceptualization of satisfaction. Scores on the general satisfaction subscale will be used as the outcome variables and other subscale scores will be used as predictor variables.

##### Reliability

The test-retest reliability, or reproduciblity, of a survey instrument refers to its ability to consistently measure all domains of diseases-specific functional status over time in a stable cohort of patients [98-100]. The more reliable, or reproducible, a measure is, the smaller the required sample size for meaningful applications of the questionnaire. Exploratory factor analysis will be used to measure reliability (using Cronbach's coefficient alpha [101]). The existing scales from the Cockburn and PAMS instruments as well as constructs derived from the focus groups will be used as the theoretical basis for naming factors revealed by this factor analysis.

##### Mammography Satisfaction Variation

We will use descriptive statistics to explore the extent to which the mammography experiences and mammography satisfaction of American Indian/Alaskan Native women vary. Frequencies and percentages will be used to summarize categorical demographic information of the participants surveyed. The primary outcome will be the global satisfaction score on the AIMS with secondary outcomes being the derived AIMS subscales. AIMS subscales will be derived from barriers cited by focus group participants and confirmed through factor analytic procedures conducted during the AIMS pilot test. Projected subscales will include service accessibility, results reporting procedures, staff interpersonal skills, perceived technical competence of staff, and global satisfaction. To model the global AIMS scores on individuals adjusting for individual factors (age, race/ethnicity, etc.), linear regression models will be developed. Similar models will be developed for AIMS subscale scores.

Using global patient satisfaction as the primary outcome variable, we also then conduct comparison of AIMS survey characteristics between women who sought services within and outside of the Indian Health Service system. This will be done by averaging the global AIMS scores for participants with an Indian Health Service-sponsored mammogram and those with a non-Indian Health Service-sponsored mammogram. Similar averaging will be done for each AIMS subscale to obtain global AIMS subscale scores.

##### Sample Size Justification

Specific Aim 3 of this study is to assess the difference in satisfaction of mammogram experiences between American Indian women who had mammogram in facilities affiliated to the Indian Health Service (IHS) and American Indian women who had mammogram in facilities not affiliated to the HIS. The sample size is justified based on the global satisfaction score of the questionnaire developed in Specific Aim 2 for satisfaction regarding the mammogram experience. Assuming the total score follows normal distributions and a frequency distribution of 10% (women with mammograms from IHS facilities) and 90% (women with mammograms from non-IHS facilities) in these two groups, a total sample size of 300 American Indian women, expected to be 30 from IHS-affiliated and 270 from non-IHS-affiliated institutions, will provide 80% power at 0.05 significance level to detect an effect size of 0.54 in the total score of satisfaction according to a two-sided two-sample t-test.

## Discussion

Mortality rates for breast cancer are rising in American Indian/Alaskan Native population and mammography rates are lowest in AI/AN. Understanding the mammography experiences and satisfaction with mammography of a heterogeneous AI/AN population is crucial to developing effective interventions to promote routine mammography utilization among this at-risk population. Over half of all AI/AN no longer live on reservations, but instead live in urban centers or, less so, in rural areas in heterogeneous groups [102]. To date, the AI/AN population in general has been understudied and the truly heterogeneous groups have in particular been left out of the literature. No systematic assessment of mammography experiences and satisfaction in a heterogeneous group that includes urban, rural, and reservation AI/AN has yet been undertaken. The data generated from this study can immediately be used to enhance the lack of data available on mammography satisfaction in the AI/AN population. The literature has shown variation in patient satisfaction studies [70,74]. We expect to find similar variations in the mammography experiences of American Indian/Alaskan Native women. The addition of culturally appropriate questions fueled by the focus groups (that were beyond those included in existing patient satisfaction instruments), will also likely uncover new variations not unearthed by prior research. The uniqueness of this study is highlighted by the fact that we will be able to assess the extent to which patient satisfaction varies among the American Indian/Alaskan Native population and whether satisfaction varies significantly when mammography services are accessed by American Indian/Alaskan Native women within or outside of Indian Health Service facilities. Any differences uncovered will be the catalyst for further study of American Indian/Alaskan Native women and future interventions to improve their mammography experience and alleviate mammography screening disparities.

## Competing interests

The authors declare that they have no competing interests.

## Authors' contributions

KKE, KAG, and EFE contributed to the design of the study and identified the research questions and hypotheses. KKE and FNM were responsible for obtaining ethics approval. BJG planned the statistical analyses. KKE, FNM, KAG, and CMD oversee the study implementation. BF and EFE serve as a scientific reviewer for the project. SB and SJ are part of the study implementation team. All authors have read, revised, and approved the final manuscript.

## Pre-publication history

The pre-publication history for this paper can be accessed here:

http://www.biomedcentral.com/1472-6874/10/34/prepub
